# Math Self-Efficacy and STEM Intentions: A Person-Centered Approach

**DOI:** 10.3389/fpsyg.2018.02033

**Published:** 2018-10-23

**Authors:** Li Lin, Taehun Lee, Lori Anderson Snyder

**Affiliations:** ^1^Department of Psychology, The University of Oklahoma, Norman, OK, United States; ^2^Department of Psychology, Chung-Ang University, Seoul, South Korea

**Keywords:** math self-efficacy, STEM, person-centered approach, latent profile analysis, mathematics education

## Abstract

Research pertaining to STEM interest and persistence continues to be a top priority in the educational research arena. The current study employed a person-centered approach to examine the impact of math self-efficacy and various distal predictors, such as individuals’ demographic information, beliefs about math, and social group identification, on STEM interest and intentions. Specifically, we conducted a latent profile analysis (LPA), thereby inferring three homogeneous subgroups of individuals or latent classes from their response patterns on the 18-item sources of math self-efficacy measure. Our analyses showed that individuals’ ethnicity, implicit theories of math ability, and other group orientation were predictive of class membership (Mastery, Moderate, and Unconfident). We also found that there were significant differences in interest in STEM subjects, interest in STEM activities, individuals’ majors, and retention grade point average across the three latent classes. Our findings support the importance of math self-efficacy in choice of major as well as overall academic performance regardless of whether a student is in a STEM field or a non-STEM field.

## Introduction

The fact that racial and ethnic minorities^1^ are underrepresented in science, technology, engineering, and mathematics (STEM) fields is well-documented (National Science Board, 2010; Chen, 2013; National Science Foundation’s National Center for Science and Engineering Statistics, 2013). Individuals who identify as Black, Hispanic, and Native American each constitute a disproportionately small percentage of science and engineering degree recipients and jobholders in STEM fields (National Science Foundation’s National Center for Science and Engineering Statistics, 2013). Chen (2013) reported that among students who started their bachelor’s degree in 2003 and 2004, approximately 10% of Asian students left STEM by dropping out of college compared to about 20% of White students, 23% of Hispanic students, and 29% of Black students. Researchers suggested that the difference between Asian students and students of other minority groups in STEM representation may be due to the fact that Asian students are often better prepared in high school mathematics (Berkner and Choy, 2008).

According to Chen (2013), mathematics education is fundamental to persistence in STEM. For example, the report found that proportionally more STEM leavers (than STEM persisters) did not earn math credits in their first year of college at both the bachelor’s and the associate’s degree levels. Additionally, compared with students who took calculus or other advanced math courses during their first year of college, those who took introductory math courses had a higher probability of switching out of STEM majors (Chen, 2013). Students with high grades in middle school mathematics were shown to have increased likelihood of being attracted to STEM at the end of high school (Sadler et al., 2012). General research also converges in agreement that math achievement is directly associated with entrance, persistence, and success in STEM (e.g., Tyson et al., 2007; Rask, 2010; Wang, 2013). Given the critical role of math training to entrance and retention in STEM fields, additional research should further investigate the antecedents and outcomes of students’ attitude and perception toward math.

### Theoretical Framework: Social Cognitive Career Theory

Based on the fundamental assumption of social cognitive theory that behaviors are derived from the interplay of person predispositions and the environment (Bandura, 1986), Lent et al. (1994) proposed social cognitive career theory (SCCT), which outlined the interactive variables and paths that are involved in a person’s decision to select and enter a career field. According to the theory, people’s beliefs about their capability to attain career goals are partly determined by their background such as racial/ethnic group, socioeconomic status, and early learning experiences. Individuals are more likely to make educational and occupational choices that are consistent with their self-efficacy and interests, particularly if accompanied by favorable environmental or contextual variables (Lent et al., 1994; Brown and Lent, 2016).

Social cognitive career theory regards self-efficacy as the central variable that predicts career outcomes, such as interests, intentions, and goals. This association between self-efficacy and academic/career outcomes has been supported by research in educational psychology and counseling psychology (e.g., Gainor and Lent, 1998; Britner and Pajares, 2006; Estrada et al., 2011). That is, research generally supports that how efficacious a person feels toward his or her academic performance and chosen career path influences the individual’s actual achievement. In a longitudinal study examining the achievement and persistence of engineering students, Lent et al. (2015) found that self-efficacy was the most reliable direct predictor of academic satisfaction and intended persistence across time. In addition, academic self-efficacy had a significant effect on outcome expectations and interests in a sample of ethnic minority students in biological science and engineering (Byars-Winston et al., 2010). Similarly, Flores et al. (2014) found support for intercorrelated relationships among self-efficacy, interests, and outcome expectations in a sample of White and Hispanic engineering students.

Bandura (1986) suggested that self-efficacy can be traced to four sources: past performance, vicarious learning, verbal persuasion (sometimes referred to as social persuasion) and physiological arousal. Past performance, or the mastery of experiences, shapes a person’s present self-efficacy beliefs by relying on past success and mastery of tasks. Vicarious learning refers to the development of efficacy through social comparisons and observation of others. Verbal or social persuasion refers to encouragement and influence from salient others. Physiological arousal refers to negative moods and emotions experienced in association with a task. These components jointly define self-efficacy, which according to SCCT, is the result of a person’s belief system, personality characteristics and background context such as socioeconomic status.

### Personal and Background Contextual Variables

Lent et al. (1994) suggested that demographic variables (e.g., race and gender) often evoke systematic sociocultural reactions from the environment (e.g., racial stereotypes) which shape people’s self-efficacy beliefs. For example, one meta-analysis found that females displayed higher language arts self-efficacy while males displayed higher math self-efficacy (Huang, 2013). The gender differences in math self-efficacy appeared to have emerged in late adolescence, with the largest difference in samples that are 23 years or older (Huang, 2013). In terms of race/ethnicity, Schweinle and Mims (2009) found that in predominantly Black/African American classrooms, White students displayed higher self-efficacy. However, racial/ethnic differences in self-efficacy may be due to socioeconomic status (Graham, 1994; Pajares and Schunk, 2001).

Phinney (1992) suggested that differences in the extent to which individuals are connected to or involved with their ethnic or cultural groups could lead to meaningful and practical outcomes. Later studies found that ethnic identity was predictive of academic adjustment (Fuligni et al., 2005) and performance (Supple et al., 2006) in ethnically diverse samples. According to Phinney (1992), ethnic identity is a form of social group identification that constitutes a large part of a person’s self-concept (also see Tajfel, 1978, 1981). Ethnic identity is defined as the recognition of and attachment to one’s ethnic group, which includes both emotional and behavioral components (Phinney, 1992). Researchers have also argued that a larger proportion of the construct (i.e., ethnic identity) is defined by the attitudinal component rather than the behavioral component (e.g., Phinney, 1992; Roberts et al., 1999; Phinney and Ong, 2007).

Ethnic identity plays a crucial role in learning and developmental experiences for ethnic minority individuals (e.g., Kerpelman et al., 2008). Specifically, ethnic- or cultural-related conflicts may result in maladaptation, higher levels of stress (e.g., Smedley et al., 1993; Gillock and Reyes, 1999), and problems with academic persistence (Hernandez and Lopez, 2004). In line with SCCT’s proposition on the relationship between person variables and self-efficacy, stereotype threat theories (see Spencer et al., 2016 for a review) suggest that a person’s ethnic background and preexisting beliefs about ethnic group membership can influence his or her performance, which then influence the person’s belief about his or her capabilities and interest in a particular domain. For example, Davies et al. (2005) suggested that low enjoyment and low confidence may explain why women who have experienced stereotype threat reported lower levels of interest in STEM fields and lower aspirations to be in leadership positions. Similarly, for individuals who are highly identified with their ethnic group, stereotype threat theories explained why certain ethnic minority groups are underrepresented in STEM (Walton and Spencer, 2009).

In addition to ethnic identity, Phinney (1992) also suggested that a person’s attitude and feelings toward ethnic groups other than one’s own are a critical part of a person’s overall social group identification. Other group orientation (OGO), a subcomponent of the original ethnic identity measure (Phinney, 1992), assesses the extent to which a person feels connected to ethnic group(s) other than his or her own. Research has suggested that a person’s ethnic identity, including his or her openness to other groups, may be related to psychological outcomes, such as self-esteem, feelings of belonging, stress, and well-being (e.g., Cokley and Chapman, 2008; Syed and Azmitia, 2009; Chance, 2013). In addition, OGO was found to be positively related to self-efficacy in a path model using a sample of underrepresented students in biological science and engineering (Byars-Winston et al., 2010).

Social cognitive career theory suggests that people’s basic values and beliefs can influence their learning experiences and therefore affect the perceptions of their ability. Findings from a large number of studies have supported the association between implicit theories of intelligence and students’ academic effort and performance (e.g., Dweck, 1996, 2002, 2012). Dweck and colleagues (e.g., Dweck et al., 1995; Chiu et al., 1997) hypothesized that people enact different attitudes and behaviors based on their perceptions regarding the malleability of attributes such as intelligence, ability, and personality. Dweck asserted that individuals who believe that these attributes are malleable tend to exert more effort and are more mastery-oriented compared to those who believe that the attributes are fixed. This idea has been further supported in educational research (e.g., Stipek and Gralinski, 1996; Bråten and Strømsø, 2004; Shively and Ryan, 2013), in which malleable beliefs were associated with higher performance and fixed beliefs were associated with lower performance. In addition, implicit theories of science ability were found to predict science self-efficacy in a number of studies (e.g., Chen, 2012; Chen and Usher, 2013).

According to SCCT, the process by which a person makes academic-related or career-related decisions can be divided into the initial expression of interest and the actions that one takes to further develop the interest. The paths from self-efficacy to interest and choice have generally been supported in previous research (e.g., Lent et al., 2008; Sheu et al., 2010). In addition to the choice model of SCCT (i.e., the paths from self-efficacy and outcome expectations to interest and choice), research has also supported the performance model of SCCT (paths from self-efficacy to performance). For example, Wright et al. (2013) found that academic self-efficacy predicted students’ academic success and their likelihood of remaining in college beyond their first semester, after controlling for gender, ethnicity, first-generation status, and prior performance. Because of its well-supported framework, the SCCT has been widely used in the research of underrepresented groups in STEM (Fouad and Santana, 2017).

## The Present Study

Utilizing SCCT as the theoretical framework, the current study investigated relationships among person and background contextual variables, math self-efficacy, and academic outcomes. More specifically, targeting issues involving the underrepresentation of ethnic minority students in STEM, the current study operationalized the outcome domain to be STEM interest and choice. Previous studies that utilized the SCCT framework have all adopted a variable-centered approach (e.g., path models). To our knowledge, none has attempted to investigate the issue using a person-centered approach.

We were interested in investigating the issue from a person-centered approach for two main reasons. First, under a variable-centered approach, factor structures of math self-efficacy varied across studies (e.g., Lent et al., 1991, 1996). The lack of a consistent factor structure across samples suggested that there may be heterogenous groups that are unknown to the researchers, which can be identified using a person-centered approach. Second, we the authors believed that a person-centered approach would be appropriate for the current study because the approach would directly address our research questions. Masyn (2013) and Horn (2000) argued that variable-centered approach and person-centered approach should be viewed as complementary in the sense that variances that are accounted for in a variable-centered approach can also be accounted for in a person-centered approach, and vice versa. That is, the differences between the two are in the setup of research questions and interpretation of results. Different models capture different aspects of the same reality and thus the criteria for model selection such as parsimony, theoretical consistency, description and prediction should be weighted according to the scientific goals for the particular application (Bauer and Curran, 2003, p. 388–391). We the authors also believe that it is important to consider the research question and scientific goal of the given application when choosing which approach to take. Most importantly, we considered the fact that parameters that would directly address our research questions are estimated in the latent profile analysis (LPA) models. Masyn (2013) suggested one should consider the consequences (e.g., anticipated model results and implications) and nature of the sample (e.g., whether the sample is heterogeneous) when deciding on the approach. Based on these recommendations, we considered potential model fit issues in variable-centered approach and heterogeneity (due to race/ethnicity) in the current sample, and decided that a person-centered approach would be more appropriate for the current study.

In addition to understanding the number and characteristics of the different classes that math self-efficacy would generate, we were interested in understanding the predictors (e.g., demographics) of these classes, that is, the variables that determine the likelihood of individuals being assigned to each of the classes. Under the scope of SCCT, both person and contextual variables should be associated with math self-efficacy. Given findings in past research regarding gender differences in interest (e.g., Su et al., 2009) and career self-efficacy (Huang, 2013), as well as the underrepresentation of women in STEM fields (e.g., National Science Foundation’s National Center for Science and Engineering Statistics, 2013), we expected that proportionally fewer women would be grouped into the class that is characterized by high accomplishments in math.

The percentage of STEM degrees conferred to individuals differs substantially by race/ethnicity (National Science Board, 2010). The National Science Foundation’s National Center for Science and Engineering Statistics (2013) does not consider Asians to be underrepresented in STEM. In contrast, national reports regarding participation in STEM (e.g., Chen, 2013; National Science Foundation’s National Center for Science and Engineering Statistics, 2013) sometimes categorize Native Americans as “other” due to insufficient sample size. In terms of general educational achievement, Native Americans are less likely to graduate from high school and college than other racial/ethnic groups (Aud et al., 2010; Stetser and Stillwell, 2014). The performance gap in mathematics between Native American and White students has also been confirmed in research (e.g., Marchand et al., 2005; National Center for Education Statistics, 2016). In the current study, we thus expected that relative to White and Asian individuals and proportionate to the subgroup sample size, a lower number of Native Americans would be categorized into the class that is characterized by high accomplishments in math.

Although the literature has been supportive of the relationship between ethnic identity and psychosocial and performance outcomes of students in general, some studies point to the contrary. For example, while Schwartz et al. (2007) found that ethnic identity was positively related to self-esteem and academic grades, O’Brien et al. (2011) found that ethnic identity centrality was not significantly correlated with GPA or feelings of belonging to the university. Moreover, Byars-Winston et al. (2010) did not find a significant coefficient from ethnic identity to academic self-efficacy in a path model. Given the mixed findings, we sought to investigate the effect of ethnic identity on the latent classes that are identified by the LPA model. On the other hand, given that Byars-Winston et al. (2010) found a positive relationship between OGO and engineering and biology self-efficacy, we anticipated that OGO would be positively associated with the class that is characterized by high accomplishments in math.

In addition to ethnic identity and OGO, an overwhelming amount of research has indicated that a more malleable or less fixed belief toward human attributes in a given domain is associated with performance in that domain (see Dweck, 2012 for a review). As mentioned previously, implicit theories of science ability was found to predict science self-efficacy in a number of studies (e.g., Chen, 2012; Chen and Usher, 2013). Thus, we hypothesized that a strong belief that math ability is fixed would be negatively associated with the class that is characterized by high accomplishments in math.

Moreover, we were interested in examining how membership in each of the classes would predict interest in STEM-related activities, interest in STEM subjects, the probability of choosing a STEM major, and general retention GPA. According to the SCCT, academic self-efficacy determines interest and choice behavior in corresponding domains. Past research has also supported the paths from academic self-efficacy to interest, intention, and choice (e.g., Flores et al., 2014; Lent et al., 2015). Thus, we expected that the class that is characterized by high accomplishments in math would be associated with higher levels of interest in STEM-related activities, higher levels of interest in STEM subjects, and higher probability of having a STEM major. Moreover, past research has indicated that math ability was highly correlated with general performance in college. Adjusting for range restriction, the correlation between the math section of Scholastic Aptitude Test (SAT) and first-year GPA was 0.49 (Kobrin et al., 2008). Academic self-efficacy was also predictive of performance in addition to interest and choice (Wright et al., 2013). Thus, we expected that the class that is characterized by high accomplishments in math would be associated with higher overall retention GPA. Supplementary Figure 1 shows a depiction of the current model.

## Materials and Methods

### Participants and Procedures

Participants that are included in the current study were respondents of a large-scale, five-year survey project that took place at a large research university in the south-central region of the United States. For this large-scale project, researchers obtained students’ email addresses from the university’s admissions office, and invited them to participate in an online survey that took about 45 minutes to complete on average. Students were compensated with a $20 gift card for completing the survey. In addition, researchers were able to access students’ academic record, including information on academic major and GPA, if permission was granted in the survey.

Given that data are collected continuously, we were only able to use a subset in the current study. The current participants were those who completed the survey during the Spring 2014 semester. Although 810 students started the survey (i.e., clicked on the survey link), only 658 (59.9% females and 40.1% males) provided responses to the measures that were used in the current study. In addition, 457 students (average age = 21) gave permission to the researchers to access their academic information.

In the survey, participants were given the option to self-report multiple racial/ethnic groups, including Black or African-American, Asian, White, Native American or Alaska Native, Native Hawaiian or other Pacific Islander, and Hispanic or Latino. The three ethnic groups that were most frequently selected were Asian, White, and Native American.^2^ Since the participants were allowed to check more than one race/ethnicity, we regarded Asian, White, and Native American as independent categorical variables with binary information (1 = participants checked the box; 0 = participants did not check the box). Participants who reported themselves as White or a combination of White and other race/ethnicity consisted of about 58% of the total sample, while those who reported themselves as Asian or a combination of Asian and other race/ethnicity consisted of about 31% of the total sample. Those who reported themselves as Native American or a combination of Native American and other race/ethnicity consisted of about 40% of the total sample. Majors and fields that were represented in the sample include humanities, business, journalism, chemistry, biology, and engineering.

### Measures

#### Sources of Math Self-Efficacy

We used 18 items from the Sources of Math Self-efficacy measure found in Usher and Pajares (2009) as the indicators of the LPA model. Sample items include “I have always been successful with math,” “People have told me that I have a talent for math,” and “I start to feel stressed out as soon as I begin my math work.” Items such as “Seeing adults do well in math pushes me to do better” in the original measure were not used because of the inappropriateness for the current sample (i.e., adult students). Participants rated the items using a scale from 1 (strongly disagree) to 6 (strongly agree).

#### Implicit Theories of Math Ability Measure

Implicit theories of math ability (ITMA) was measured with an 8-item instrument that was modified based on Dweck’s (1999) measure of implicit theories of intelligence. Similar to Chen and Usher (2013), we modified the scale to reflect “math ability” instead of general intelligence. Higher scores indicate a stronger belief that math ability is fixed. Participants rated the items using a scale from 1 (strongly disagree) to 6 (strongly agree). A sample item was “You can learn new things, but you can’t really change your basic math ability.” The items framed in the opposite direction (e.g., “No matter how much math ability you have, you can always change it quite a bit”) were reverse-coded. The Cronbach’s alpha for the 8-item ITMA was 0.90.

#### Multigroup Ethnic Identity Measure and Other Group Orientation

Ethnic identity was measured using a revised version of MEIM (Roberts et al., 1999). The instrument consisted of 12 items that assessed people’s feelings of connection and belonging to one’s own ethnic group and the level of involvement and exploration of ethnic histories and events that are specific to the group. Participants were first asked to select one ethnic group with which they most identified. The participants were then asked to rate the 12 items on a scale from 1 (strongly disagree) to 4 (strongly agree). Sample items were “I have spent time trying to find out more about my ethnic group, such as its history, traditions, and customs,” “I have a clear sense of my ethnic background and what it means for me,” and “I think a lot about how my life will be affected by my ethnic group membership.” Higher scores on this measure indicate that the person has positive attitudes, such as pride and belonging, toward his or her ethnic group membership and is highly involved in seeking out information relating to the ethnic group. The Cronbach’s alpha for the measure was 0.90.

The original MEIM contains OGO as a subcomponent (Phinney, 1992). However, Phinney (1992) stated that one’s attitudes toward other ethnic groups should be independent of those toward one’s own ethnic group, despite the fact that orientation to other groups is a part of one’s overall social identity. In the current study, ethnic identity and OGO were approached as separate variables. OGO was measured with the subcomponent of the original MEIM (Phinney, 1992). The measure consisted of six items, two of which were reverse-coded. Sample items were “I like meeting and getting to know people from ethnic groups other than my own,” “I sometimes feel it would be better if different ethnic groups didn’t try to mix together (reverse-coded),” and “I am involved in activities with people from other ethnic groups.” Participants rated the items on a scale from 1 (strongly disagree) to 4 (strongly agree). Higher scores indicate higher orientation and more welcoming attitude toward other groups. The Cronbach’s alpha for the measure was 0.70.

#### Interest in STEM Subjects

The measure for interest in STEM subjects was adopted from Lent et al. (2001). Participants were asked to indicate their interest in STEM subjects on a scale from 1 (Strongly Dislike) to 5 (Strongly Like). The list of STEM subjects included Statistics, Chemistry, Physics, Basic Math, Computer Science, Biology, Advanced Math, and Engineering. Higher scores indicate that participants have higher interest in STEM subjects. The Cronbach’s alpha for interest in STEM subjects was 0.83.

#### Interest in STEM Activities

The measure for interest in STEM-related activities was adopted from Lent et al. (2001). Participants were asked to indicate their interest in STEM-related activities on a scale from 1 (Strongly Dislike) to 5 (Strongly Like). The list of STEM-related activities included “solving practical math or science problems,” “reading articles or books about scientific issues,” and “solving computer software problems.” Higher scores indicate that participants have higher interest in STEM-related activities. The Cronbach’s alpha for interest in STEM activities was 0.87.

#### Current Major

As one of the outcome variables, students’ academic major was coded into STEM categories. Prior to coding participants’ major, we assigned each major at the university a STEM code. Majors that are commonly considered as a part of the STEM curriculum or are highly related to STEM curriculum were given a numeric code of 1. The majors that were coded as 1 include all majors in the engineering college, mathematics department, chemistry department, biology department, physics department, and computer science department. Majors that are associated with social and behavioral sciences and/or require some science, technology, engineering, or mathematics training, but are not traditionally considered as a part of STEM, were assigned a numeric code of 2. For example, psychology and anthropology were assigned a numeric code of 2. Majors that are lowest in STEM content, such as those in the fine arts college, journalism college, English department, and foreign language department were given a numeric code of 3.

The process of coding began with consultation of a variety of STEM taxonomies, such as those listed by the National Science Foundation, National Institutes of Health, and Institutional Research and Report office of the university in which the study was conducted. A team of researchers, including two of the authors of the current study and two additional graduate research assistants, created the Major-to-STEM Code key by sorting every major that was offered at the university into the three STEM categories. Then, two undergraduate research assistants utilized the key, and independently coded participants’ majors into STEM codes. Because of minor discrepancies in the institution’s information storage system (e.g., two distinct majors were referred to by the same or highly similar names), the two coders agreed on 88% of the majors in this process. In the final step, the two undergraduate research assistants and two graduate research assistants discussed the majors in question and reached consensus. In the sample to which the final model was fitted, 244 of the participants were assigned a STEM code of 1, 81 of the participants were assigned a STEM code of 2, and 132 of the participants were assigned a STEM code of 3.

#### Retention GPA

Participants’ retention GPA as of Fall 2014 (inclusive of Spring 2014 class grades) was retrieved simultaneously with their declared major. Retention GPA is a cumulative summation of grades from all classes that a student has taken on a 4-point scale. It was used by the University in administrative policies and thus was regarded as a justified comparative metric across students of different academic majors. The average retention GPA for the students that were included in the final model was 3.28.

### Analysis

#### Conducting Latent Profile Analysis

In the first step, we conducted latent profile analyses using the 18 items in the math self-efficacy scale. Based on recommended procedures in Masyn (2013), we fitted a series of models with a varying number of latent classes. We selected the model that provides not merely adequate representation of the data but also substantively meaningful interpretation of the identified latent classes. We assumed that the covariances among the indicators within a class were zero. We allowed the variances to differ across classes, thereby allowing some classes to be more heterogeneous than other classes with respect to the responses to the observed variables (Hagenaars and McCutcheon, 2002). Model parameters were estimated by EM algorithm, and the associated standard error estimates were obtained by the robust or the sandwich estimator (Collins and Lanza, 2010).

Although there are no ‘golden rules’ to judge the fit of a latent profile model, researchers have recommended several criteria to judge the goodness of a model solution. Common model selection criteria such as Akaike Information Criterion (AIC) and Bayesian Information Criterion (BIC) were used to compare models. Lo-Mendell-Rubin Likelihood Ratio Test (LMR-LRT; Lo et al., 2001) can be used to judge the statistical improvement obtained by adding classes to the model one at a time. Furthermore, the substantive quality of the latent profile model solution can be assessed by entropy, proportion of the smallest class, class homogeneity, class separation, and posterior probability (see Hagenaars and McCutcheon, 2002; Marsh et al., 2009; Masyn, 2013 for more details). We followed the recommendations in Masyn (2013) for the evaluation of model fit and substantive quality in the evaluation and selection of the model.

#### Latent Class Model With Covariates and Outcomes

Adding covariates in the model to predict the latent class membership essentially involves conducting a multinomial logistic regression analysis to predict probability for each category of the dependent variable. In multinomial logistic regression, one category is designated as the reference/baseline category, and the effects of covariates for each latent class are estimated in comparison to the reference/baseline class. The estimated intercepts and regression coefficients are transformed into odds and odds ratios, respectively, by using exponential function. The exponentiated intercept represents the odds of membership in one class relative to the reference class when all covariates are equal to 0. The exponentiated regression coefficient of a covariate represents the change in odds of membership in one class relative to the reference class when the covariate is changed by one unit, holding other covariates constant.

In the analysis of covariates for the current LPA model, we examined how various predictors, including gender, Native American ethnicity, MEIM, OGO, and ITMA, would affect the probability of membership in each of the classes that was identified by the LPA model. However, when all of these variables were entered into the model, only Native American, ITMA, and OGO emerged as significant predictors of class membership. Following the suggestions in Collins and Lanza (2010), we tested the interaction among these three variables: Native American by ITMA, Native American by OGO, and OGO by ITMA. Among the three interaction terms, only OGO by ITMA was found to be a significant predictor. Thus, the final model included four covariates: (1) Native American, (2) OGO, (3) ITMA, and (4) OGO by ITMA. The continuous variables (i.e., OGO and ITMA) were standardized before they were included in the model.

In the analysis of outcomes for the current LPA model, we examined how class membership predicts the outcome variables of interest. Masyn (2013) referred to this as the “three-step approach” where classes generated by the LPA model can be utilized as predictors for outcome variables. In the first step, participants were categorized into latent classes based on their highest posterior probability of being in a given class. In the second step, one would create a new data file that contains the class assignments. In the last step, one would regress the outcome variables on the most likely class membership. We followed these three steps and regressed interest in STEM subjects, interest in STEM-related activities, major (coded in terms of STEM categorization), and retention GPA on participants’ latent class membership. In the regression model, STEM code was treated as a nominal variable. The STEM code of 2 was used as the reference. We used full information maximum likelihood (FIML)^3^ in Mplus to account for the missing data in students’ major and retention GPA. All statistical analyses were conducted in Mplus Version 7.11 (Muthén and Muthén, 1998–2011). Supplementary Figure 2 displays the final list of predictors and outcomes.

## Results

The descriptive statistics and correlations for the predictor variables are presented in Supplementary Table 1. The inter-item correlations for the sources of math self-efficacy measure are presented in Supplementary Table 2.

### Results of Latent Profile Analysis

To decide on the number of classes, we compared models with between one and four classes using a variety of statistical fit indexes, including information criteria, LMR-LRT, entropy, and the size of the smallest class. Compared with other models, the AIC, BIC and adjusted BIC were lower for the three-class model (Supplementary Table 3). The LMR-LRT also indicated that there was no improvement in fit for the inclusion of one more class to the three-class model (i.e., *p*LMP = 0.50). Moreover, the size of the smallest class in the four-class model was zero. Entropy was the highest for the three-class model, which indicates that individuals were classified into latent classes more accurately in the three-class model than in the other models. More importantly, the three-class model offered much clearer substantive interpretability than the four-class model. Thus, we selected the three-class model as the final model. The profiles for the three-class model are presented in Supplementary Figure 3.

#### Mastery (*n* = 252, 38%)

The Mastery group was characterized by high estimated means for items 1, 2, and 4 through 12 (μ = 5.25, 5.34, 5.31, 5.42, 4.91, 5.07, 5.12, 5.20, 5.18, 5.24, and 5.15, respectively) and low estimated means for items 3 and 13 through 18 (μ = 1.39, 1.77, 2.10, 1.70, 1.49, 1.34, and 1.34, respectively). The mean posterior probability of a member classified in the Mastery group to belong to the Mastery group was 0.99, to belong to the Moderate group was 0.01, and to belong to the Unconfident group was 0.00.

#### Moderate (*n* = 302, 46%)

The Moderate group was characterized by its mediocre scores on items 1 through 6 (μ = 3.91, 3.90, 2.87, 4.12, 4.42, and 3.70, respectively), items 7 through 12 (μ = 3.62, 3.32, 3.39, 3.43, 3.43, and 3.43, respectively), and items 13 to 18 (μ = 3.33, 3.57, 3.26, 2.97, 2.72, and 2.62, respectively). The mean posterior probability of a member classified in the Moderate group to belong to the Mastery group was 0.01, to belong to the Moderate group was 0.99, and to belong to the Unconfident group was 0.00.

#### Unconfident (*n* = 104, 16%)

The Unconfident group was characterized by low estimated means for items 1, 2, and 4 through 12 (μ = 2.25, 1.88, 2.83, 2.72, 1.72, 1.75, 1.45, 1.48, 1.38, 1.41, and 1.49, respectively) and high estimated means for items 3 and 13 through 18 (μ = 4.45, 5.26, 5.21, 5.20, 4.84, 4.59, and 4.48, respectively). The mean posterior probability of a member classified in the Unconfident group to belong to the Mastery group was 0.00, to belong to the Moderate group was 0.01, and to belong to the Unconfident group was 0.99.

### Results of Latent Class Model With Covariates and Outcomes

In the second part of the analysis, we added four covariates (Native American, OGO, ITMA, and OGO by ITMA) to the three-class model. The model information for the final model was as the following: number of free parameters = 118; Log likelihood = -16390; AIC = 33017; BIC = 33542; sample-size adjusted BIC = 33167; and entropy = 0.97. The class prevalence between the model with covariates and the model without covariates remained highly similar (Mastery = 247, 39%; Moderate = 285, 45%; Unconfident = 101, 16%). In other words, the two models have no practical difference in the estimated class posterior probabilities for each individual. Minor differences could be due to the reduction of sample size in the model with covariates. Because of missing data in the covariates, the sample size in the model with covariates changed from 658 to 633. The likelihood ratio test statistic comparing the two models [using the -2 (*L_1_*-*L_2_*) formula] was 1478, with *df* = 8, and *p* < 0.001, indicating that the four covariates were statistically significant.

#### Predictors of Latent Classes

The results for the effects of the predictors of the latent classes were estimated as the odds of (1) membership in the Unconfident group relative to the Moderate group, (2) membership in the Mastery group relative to the Moderate group, and (3) membership in the Unconfident group relative to the Mastery group.

##### Unconfident (moderate as reference)

The odds coefficients were significant for the intercept and two of the four predictors—Native American and ITMA (see Supplementary Table 4). The odds ratios for OGO (odds ratio = 1.36) and OGO^∗^ITMA (odds ratio = 1.09) were found to be non-significant. The estimate of the intercept indicated that when all predictors were equal to 0, the odds of membership in the Unconfident group relative to the Moderate group was 0.17. It should be noted that all continuous predictors were standardized, such that the mean of each variable equaled 0. Therefore, the intercept estimate of 0.17 suggested that, when the “Native American” box was not selected and the scores of ITMA and OGO were at their means, the odds of being in the Moderate group was almost six times the odds of being in the Unconfident group (the reciprocal of 0.17 = 5.88).

As shown in Supplementary Table 4 and Supplementary Figure 4, for one unit increase in Native American, the odds of membership in the Unconfident group relative to the Moderate group increased by a factor of 2.58. That is, participants who indicated “Native American” as (a part of) their ethnicity, were (2.58 times) more likely to belong to the Unconfident group, whereas non-Native American participants were more likely to be in the Moderate group when all other variables in this model were held constant.

The odds ratio was found to be significant for ITMA (odds ratio = 2.55), indicating that for one standard deviation increase in ITMA, the odds of membership in the Unconfident group relative to the Moderate group increased by a factor of 2.55. That is, participants who indicated higher agreement to the view that math ability is fixed are more likely to belong to the Unconfident group in comparison to the Moderate group.

##### Mastery (moderate as reference)

As shown in Supplementary Table 4 and Supplementary Figure 4, the estimated intercept and Native American were found to be non-significant, whereas three of the predictor terms (OGO, ITMA, and OGO^∗^ITMA) were found to be significant. In order to understand the nature of the interaction between OGO and ITMA, we plotted the impacts of ITMA on the odds of membership in the Mastery group relative to the Moderate group when OGO was one standard deviation below the mean, at the mean, and one standard deviation above the mean.

As shown in Supplementary Figure 5, when OGO was one standard deviation below the mean, for one standard deviation increase in ITMA, the odds of membership in the Mastery group relative to the Moderate group was 0.56 (β = -0.58). This value was 0.74 (β = -0.30) and 0.99 (β = -0.01) when OGO was at the mean and one standard deviation above the mean, respectively. Therefore, the impact of ITMA on the odds of membership in the Mastery group relative to the Moderate group was moderated by OGO such that given the same level of ITMA, participants were almost two times (the reciprocal of 0.56 = 1.79) more likely to belong to the Moderate group when OGO was one standard deviation below the mean. However, when OGO was one standard deviation above the mean, the odds of membership in the Moderate group and Mastery group were about one-to-one (the reciprocal of 0.99 = 1.01). This indicates that ITMA has greater impact on the odds of belonging to the Mastery group over the Moderate group when OGO is lower. Thus, the effects of ITMA may be more relevant in determining membership in the Mastery group (relative to the Moderate group) when the person has a weaker orientation to other ethnic groups.

##### Unconfident (mastery as reference)

The intercept and two of the four predictors (Native American and ITMA) were found to be significant. OGO and the interaction between OGO and ITMA were found to be non-significant. The intercept indicated that when all four predictors were equal to 0, the odds of being in the Unconfident group relative to the Mastery group was 0.19. When the participants did not indicate “Native American” as (a part of) their race/ethnicity and the scores of OGO and ITMA were at their means, the odds of being in the Mastery group was five times the odds of being in the Unconfident group (the reciprocal of 0.19 = 5.26).

As shown in Supplementary Table 4 and Supplementary Figure 4, when participants indicate that they are (part) Native American, the odds of being in the Unconfident group relative to the Mastery group increased by a factor of 2.83. That is, Native American participants (participants who checked the “Native American” box) were more likely to be in the Unconfident group than the Mastery group when the rest of the variables were held constant. This value was 3.42 for ITMA, indicating that given one standard deviation increase in ITMA, the odds of belonging to the Unconfident group was more than three times the odds of belonging to the Mastery group. In other words, higher endorsement of the belief that math ability is fixed was associated with a higher chance of belonging to the Unconfident group rather than the Mastery group.

#### Outcomes of the Latent Classes

To understand the relationship between the latent class variable and outcome variables, which include interest in STEM subjects, interest in STEM activities, declared major (STEM code), and retention GPA, we regressed these outcomes on the latent class membership variable. As explained earlier, we treated participants’ most likely class memberships provided by the three-class solution as an observed variable and created two dummy coded indicators, each of which represents membership in the Unconfident and the Mastery group, respectively. Membership in the Moderate group was used as the reference group. Full Information Maximum Likelihood (FIML) was used to treat missing data due to the denial of access to academic records such that the sample size of the model remained at 633.

As shown in Supplementary Table 5, membership in the Mastery group was associated with increases in interest in STEM subjects (β = 0.24), interest in STEM activities (β = 0.23), and retention GPA (β = 0.24). In addition, membership in the Mastery group was also associated with the odds of being in a STEM major, such that members of the Mastery group were about two times more likely to be in a STEM major than a major with some STEM emphasis (β = 0.705; odds ratio = 2.023).

In contrast, not only was membership in the Unconfident group associated with decreases in all of the continuous outcome variables (interest in STEM subjects, β = -0.38; interest in STEM activities, β = -0.34; retention GPA, β = -0.10), the odds of being in a STEM major was about one-third times the odds of being in a major with some STEM emphasis (β = -1.288; odds ratio = 0.276). Membership in the Unconfident group predicted retention GPA only marginally (β = -0.10, *p* = 0.059). Interestingly, neither membership in the Unconfident group nor membership in the Mastery group significantly predicted the odds of having a non-STEM major. This suggests that, while the latent class membership based on math self-efficacy predicted the odds of having a STEM major, it did not predict the odds of choosing a non-STEM major.

## Discussion

Research continues to point out problems in the selection and retention of various minority groups in STEM fields (Fouad and Santana, 2017). Given the disadvantage of variable-centered approach in assuming homogeneity within samples, utilizing SCCT, the present study took a person-centered approach to understand the relationship among personal/contextual variables, math self-efficacy, and STEM-related outcomes. We first conducted a LPA (with covariates) to identify subgroups of individuals who share similar response patterns to the sources of math self-efficacy measure, and then utilized the identified subgroup membership to predict interest in STEM subjects, interest in STEM-related activities, choice of STEM major, and overall retention GPA.

Results from the LPA model supported a three-class model. That is, based on their response patterns to the 18-item math self-efficacy measure, three distinct subgroups of individuals were present in the current sample. As expected, at least one of the groups, which we referred to as the Mastery group, was characterized by high means in the items that ask about past math accomplishment. The other two groups were referred to as the Unconfident group and the Moderate group.

In terms of the analysis of covariates for the LPA model, contrary to our expectation, gender did not emerge as a significant predictor after controlling for other predictors. This finding is consistent with previous research, such as Maltese and Tai (2011), who found that when other variables, such as the number of science or math classes attempted or completed in high school, were also present in the model, gender was non-significant in predicting completion of a degree in STEM. Other research also indicated that gender differences in math performance may be exaggerated by parents and teachers (Hyde et al., 2008).

Regarding our research question about the relationship between ethnic identity and the latent classes that were identified by the LPA model, ethnic identity did not emerge as a significant predictor. Despite the support for the association between ethnic identity and outcomes such as self-esteem and perceived support, the direct association between ethnic identity and academic self-efficacy has not been well-supported in previous research. For example, Byars-Winston et al. (2010) dropped ethnic identity from a SCCT path model due to the non-significant path from ethnic identity to academic self-efficacy. Moreover, some researchers suggested that ethnic identity may only be salient to ethnic minority individuals (e.g., Phinney, 1992). Thus, it is possible that the effect of ethnic identity was diminished in the current study because White individuals were included. Moreover, ethnic identity may not provide additional unique explanation when race/ethnicity was already included in the model.

The final LPA model (with covariates) identified Native American, OGO and ITMA, and the interaction between OGO and ITMA as statistically significant predictors to the latent class variable. As expected, the results suggested that participants who selected “Native American” as a component of their ethnicity had a higher chance of being in the Unconfident group. However, it is important to note that this finding does not have implications for causal relationships (e.g., being Native American causes low math self-efficacy), innate math ability (e.g., Native Americans are naturally less capable in math), or psychological tendencies (e.g., Native Americans are less confident in math relative to other groups despite an equal objective amount of resources). As noted in the SCCT model (Lent et al., 1994), race/ethnicity may reflect early developmental experiences and sociocultural interactions.

Implicit theories of math ability also emerged as a strong predictor of whether an individual would be categorized into the Unconfident group. Consistent with the bulk of Dweck’s research and Chen and colleagues’ work (Chen, 2012; Chen and Usher, 2013), our results indicated that a fixed belief in math ability was associated with higher odds of belonging to the Unconfident group. Whereas the Native American variable only predicted membership in the Unconfident group (not membership in the Mastery group relative to the Moderate group), ITMA predicted all three possible pairs of contrasts (i.e., Unconfident versus Moderate, Unconfident versus Moderate, and Mastery versus Moderate). In addition, OGO was found to be a significant predictor of the membership in the Mastery group in relation to only the Moderate group. Specifically, participants with higher OGO were more likely to be in the Mastery group relative to the Moderate group than the people with lower OGO.

More interestingly, OGO interacted with ITMA to predict membership in the Mastery group relative to the Moderate group such that the effect of one unit increase of ITMA was greater when OGO was lower. Given that a fixed view of math ability (higher ITMA) was associated with higher odds of membership in the Unconfident group and Moderate group, OGO appeared to have buffered the impact of a fixed view of math ability on math self-efficacy. That is, an individual would be more likely to belong to the Moderate group relative to the Mastery group if his or her OGO was low. However, this person would have an equal chance of belonging to Moderate group relative to the Mastery group if his or her OGO was high.

Previous studies have supported the “protective mechanism” of OGO in the relationship between discrimination and psychological well-being, such that given the same level of perceived discrimination, those with higher OGO would report higher levels of well-being in contrast to those with lower OGO (Lee, 2003). Additionally, researchers also found that a fixed belief about human characteristics can accentuate the effect of social identification on outcomes. For example, Hong et al. (2003) found that a sample of Chinese participants perceived a higher need to fulfill their traditional Chinese moral duties if they believe human morality to be a fixed entity. In other words, the impact of fixed beliefs about human characteristics would be greater if people are more identified with their own cultural or ethnic group and less identified with groups other than their own. To our knowledge, however, the current study was the first study to investigate the interaction effect of ITMA and OGO.

The generated three-class membership significantly predicted four STEM-related outcomes: interest in STEM subjects, interest in STEM activities, retention GPA, and the odds of being in a STEM major. The findings were consistent with SCCT’s proposition regarding the relationship between self-efficacy and interest and choice (e.g., Lent et al., 2008; Sheu et al., 2010). More importantly, we found that while the latent class membership predicted the odds of being in a STEM major, it did not predict the odds of being in a non-STEM major. In addition to outcomes in the specified domain (i.e., STEM), the latent class variable also predicted general retention GPA. The integration of results points to the importance of math self-efficacy in overall academic performance regardless of whether a student is in a STEM field or a non-STEM field.

### Limitations

Several limitations should be noted in the current study. First, similar to other studies that are conducted in university settings, current results were based on a college-aged population whereas previous studies using math self-efficacy have often been conducted with school-aged children and early adolescents (e.g., Chen, 2012; Chen and Usher, 2013). In addition, while it would be interesting to investigate math self-efficacy and STEM-outcomes on additional racial/ethnic groups, only three groups (Asian, White and Native American) were available to researchers in the current study. Second, given that this is a survey study, we could not fully eliminate the possibility of random responses due to fatigue or a lack of motivation, despite the fact that participants were compensated for their time.

Third, the current study utilized a two-step modeling approach (see Masyn, 2013) to first classify participants into latent classes based on their highest posterior probability of being in a given class, and then treat these most likely class memberships as observed variable. However, treating the most likely class memberships as observed variable can be justified because in the current data, the average posterior probability of each latent class was 0.99 for all three classes, meaning that overall uncertainty in posterior classification was extremely low. Lastly, there may be debates over the definition of STEM (i.e., what academic majors should be considered STEM). The current study was focused in general STEM fields, and did not make further distinctions between life sciences and physical sciences. Defining STEM at a granular level may yield additional insights to why certain groups are underrepresented in STEM.

### Implications and Future Directions

The current study has made several important contributions to the literature. First, to our knowledge, the current study is one of the few studies (e.g., Chen and Usher, 2013) that have examined self-efficacy using a person-centered approach. From our perspective, an alternative method should be used if the complex measure (i.e., math self-efficacy) cannot be decomposed into clean and sufficient components using the traditional technique (i.e., variable-centered approach). Furthermore, a majority of studies that involved self-efficacy used correlational designs (Usher and Pajares, 2008). A more sophisticated design, such as a longitudinal design, may be necessary to provide fruitful findings. For example, using a two-wave cross-lagged panel design, Lent et al. (2008) found that self-efficacy at one time point was related to interest, outcome expectations, and goal persistence at the second time point.

Second, the current effort speaks to the ongoing issue regarding the lack of representation of certain racial/ethnic groups in STEM. Among all racial/ethnic groups, Native Americans have one of lowest participation rates in STEM. For example, out of all majors and degrees, 4.2% of bachelor’s degrees earned by Native American students are in engineering. In contrast, 8.7% of bachelor’s degrees earned by Asian students and 5.2% of bachelor’s degrees earned by White students are in engineering (Aud et al., 2010). Yet, this group is so under-researched that it is often represented with asterisks in national reports (Shotton et al., 2013). In the current study, the effect of identification with the Native American group on the latent class variable suggests that there may be additional factors that are driving the racial/ethnic differences in STEM representation. For example, it is likely that the significant differences in math self-efficacy across racial/ethnic groups were due to systematic differences in key resources, such as the availability of advanced placement classes in high school.^4^

More recent research (e.g., O’Brien et al., 2015) has directed attention to study STEM participation from an “intersectionality” perspective (i.e., the interaction between gender and race/ethnicity). Given that Native American as an ethnic group is severely underrepresented in STEM, it may be difficult to obtain a sample of Native American women in STEM. However, it would be valuable to further investigate the role of gender in Native Americans’ career decisions. Understanding female Native American scientists’ career decisions and paths will be informative to designing interventions that focus on increasing STEM participation for socially and economically disadvantaged groups.

Lastly, the significant relationship between the latent classes and students’ choice of STEM major affirms the critical role of math in all STEM fields. Yet, the average math performance among U.S. adolescents still far short of being desirable (Desilver, 2017). Scholars suggested that math anxiety, which was defined as a feeling of tension, apprehension, or fear that interferes with math performance, may be playing a role (Ashcraft, 2002). The negative physiological and psychological reactions to math are often associated with early negative experiences with insensitive teachers (Perry, 2004). At the college level, math anxiety may be further intensified by the competitive nature of introductory STEM classes (see Seymour, 1995). Future research should investigate how the fear of math, coupled with social stereotypes, may be playing a role in the lack of representation of women and racial/ethnic minority groups in STEM.

### Concluding Remarks

Unequal STEM representation in terms of race/ethnicity remains an unresolved issue in scientific progress and innovation (National Science Board, 2014). The lack of representation also reflects a systematic deficit in math and science-related skills and knowledge in several sectors of our population, which, as the current study suggests, can be attributed to a lack of efficacy in math. While there are realistic constraints, such as economic resources, that have created barriers for these groups to pursue a science career, one of the reoccurring themes in literature (e.g., Cheryan et al., 2017) is the fact that stereotypes and normative beliefs have steered these groups away from STEM. The solution to the problem perhaps extends beyond providing economic resources. In addition, social and educational countermeasures to ensure that a person’s demographic identities (e.g., Native American identity or female identity) are not perceived as in conflict with a scientific identity are likely to have impact.

## Ethics Statement

This study was carried out in accordance with the recommendations of the institutional review board at the University of Oklahoma with written informed consent from all subjects. All subjects gave written informed consent in accordance with the Declaration of Helsinki. The protocol was approved by the institutional review board at the University of Oklahoma.

## Author Contributions

LL developed the original idea for the study, conducted the main analyses, and wrote the initial draft. TL contributed to the development of the analytic approach and provided initial reviews. LS collected the data and provided the initial reviews. All authors were involved in the revision process.

## Conflict of Interest Statement

The authors declare that the research was conducted in the absence of any commercial or financial relationships that could be construed as a potential conflict of interest.

## Footnotes

1 Research has noted some subtle differences between “race” and “ethnicity” (Cokley, 2007). These subtle differences do not influence the current study. Thus, “race” and “ethnicity” were used interchangeably throughout the study. Since participants were allowed to select multiple options to describe their race/ethnicity, in the analysis and results section, singular nouns such as “Asian,” “White,” and “Native American” were selected as names for dichotomous variables.

2 Due to the need of the longitudinal project, we intentionally oversampled the number of Asian/Asian American participants and Native American participants in the population.

3 As an alternative to full information maximum likelihood, the analysis was conducted with listwise deletion. The significance of coefficients did not differ.

4 A separate question in the survey asked participants whether advanced placement classes were available in their high school. The results of one-way ANOVA (grouping variable = race/ethnicity; participants were allowed to select only one racial/ethnic group for this analysis) indicated that the Native American group was significantly less likely to have advanced placement classes available in high school in comparison to the Asian group.
