# Supplementary material for: Math Self-Efficacy and STEM Intentions: A Person-Centered Approach
Source: Front Psychol. 2018 Oct 23;9:2033. doi: 10.3389/fpsyg.2018.02033 (PMC6206556; doi:10.3389/fpsyg.2018.02033)
Supplement: Supplementary file 1 [file Data_Sheet_1.docx]

Supplementary Material

Math Self-Efficacy and STEM Intentions: A Person-Centered Approach

Li Lin, Taehun Lee^*^, Lori Anderson Snyder

*** Correspondence:**

Taehun Lee
lee.taehun@gmail.com

# Supplementary Tables and Figures

## Tables

|  | M | SD | Range | Gender | ITMA | MEIM | OGO | Interest in STEM subjects |
| --- | --- | --- | --- | --- | --- | --- | --- | --- |
| Gender | 1.60 | 0.50 | 1.00 |  |  |  |  |  |
| ITMA | 2.79 | 0.98 | 5.00 | 0.06 |  |  |  |  |
| MEIM | 2.80 | 0.59 | 3.00 | 0.00 | -0.07 |  |  |  |
| OGO | 3.30 | 0.48 | 2.33 | 0.13^**^ | -0.21^**^ | 0.13^**^ |  |  |
| Interest in STEM subjects | 3.10 | 0.85 | 4.00 | -0.27^**^ | -0.35^**^ | -0.01 | 0.03 |  |
| Interest in STEM activities | 3.27 | 0.91 | 4.00 | -0.26^**^ | -0.31^**^ | 0.01 | 0.08^*^ | 0.82^**^ |

**Table 1.** Descriptive statistics and correlations among all predictor variables. Gender was coded as 1 = male, 2 = female. **p* < .05; ** *p* < .01

|  | M | SD | I1 | I2 | I3 | I4 | I5 | I6 | I7 | I8 | I9 | I10 | I11 | I12 | I13 | I14 | I15 | I16 | I17 |
| --- | --- | --- | --- | --- | --- | --- | --- | --- | --- | --- | --- | --- | --- | --- | --- | --- | --- | --- | --- |
| I1 | 4.16 | 1.37 | 1.00 |  |  |  |  |  |  |  |  |  |  |  |  |  |  |  |  |
| I2 | 4.13 | 1.50 | 0.81 | 1.00 |  |  |  |  |  |  |  |  |  |  |  |  |  |  |  |
| I3 | 2.56 | 1.50 | -0.65 | -0.64 | 1.00 |  |  |  |  |  |  |  |  |  |  |  |  |  |  |
| I4 | 4.37 | 1.52 | 0.66 | 0.60 | -0.49 | 1.00 |  |  |  |  |  |  |  |  |  |  |  |  |  |
| I5 | 4.54 | 1.25 | 0.75 | 0.73 | -0.58 | 0.65 | 1.00 |  |  |  |  |  |  |  |  |  |  |  |  |
| I6 | 3.85 | 1.45 | 0.74 | 0.72 | -0.57 | 0.58 | 0.79 | 1.00 |  |  |  |  |  |  |  |  |  |  |  |
| I7 | 3.88 | 1.53 | 0.68 | 0.71 | -0.53 | 0.50 | 0.67 | 0.74 | 1.00 |  |  |  |  |  |  |  |  |  |  |
| I8 | 3.71 | 1.68 | 0.65 | 0.73 | -0.52 | 0.44 | 0.61 | 0.67 | 0.80 | 1.00 |  |  |  |  |  |  |  |  |  |
| I9 | 3.77 | 1.71 | 0.67 | 0.73 | -0.55 | 0.45 | 0.63 | 0.66 | 0.76 | 0.88 | 1.00 |  |  |  |  |  |  |  |  |
| I10 | 3.77 | 1.69 | 0.69 | 0.75 | -0.56 | 0.45 | 0.65 | 0.68 | 0.76 | 0.87 | 0.89 | 1.00 |  |  |  |  |  |  |  |
| I11 | 3.80 | 1.69 | 0.71 | 0.75 | -0.56 | 0.49 | 0.65 | 0.68 | 0.77 | 0.86 | 0.84 | 0.89 | 1.00 |  |  |  |  |  |  |
| I12 | 3.78 | 1.66 | 0.72 | 0.73 | -0.57 | 0.48 | 0.64 | 0.68 | 0.72 | 0.79 | 0.79 | 0.85 | 0.89 | 1.00 |  |  |  |  |  |
| I13 | 3.04 | 1.65 | -0.58 | -0.58 | 0.66 | -0.43 | -0.56 | -0.55 | -0.52 | -0.50 | -0.50 | -0.51 | -0.52 | -0.50 | 1.00 |  |  |  |  |
| I14 | 3.27 | 1.55 | -0.52 | -0.56 | 0.60 | -0.40 | -0.50 | -0.49 | -0.49 | -0.50 | -0.49 | -0.49 | -0.50 | -0.46 | 0.76 | 1.00 |  |  |  |
| I15 | 2.97 | 1.60 | -0.60 | -0.62 | 0.67 | -0.48 | -0.57 | -0.53 | -0.50 | -0.51 | -0.51 | -0.51 | -0.53 | -0.50 | 0.82 | 0.84 | 1.00 |  |  |
| I16 | 2.70 | 1.56 | -0.60 | -0.60 | 0.67 | -0.50 | -0.56 | -0.53 | -0.50 | -0.47 | -0.47 | -0.50 | -0.51 | -0.47 | 0.76 | 0.75 | 0.85 | 1.00 |  |
| I17 | 2.49 | 1.54 | -0.58 | -0.58 | 0.66 | -0.46 | -0.56 | -0.50 | -0.46 | -0.44 | -0.48 | -0.49 | -0.51 | -0.47 | 0.71 | 0.69 | 0.78 | 0.80 | 1.00 |
| I18 | 2.42 | 1.53 | -0.56 | -0.58 | 0.64 | -0.44 | -0.58 | -0.50 | -0.47 | -0.44 | -0.46 | -0.47 | -0.48 | -0.45 | 0.72 | 0.71 | 0.81 | 0.82 | 0.85 |

**Table 2.** Inter-item correlations of Sources of Math Self-Efficacy. All coefficients are significant at *p* < .01

| Number of groups | Number of free parameters | Log Likelihood | Akaike Information Criterion | Bayesian Information  Criterion | Sample-size Adjusted BIC | Entropy | *p*LMR | Smallest class frequency (Relative frequency) |
| --- | --- | --- | --- | --- | --- | --- | --- | --- |
| 1 | 36 | -21925 | 43922 | 44083 | 43969 | - | - | 658 (1.00) |
| 2 | 73 | -18360 | 36867 | 37195 | 36963 | 0.970 | 0.000 | 320 (0.49) |
| 3 | 110 | -17143 | 34505 | 34999 | 34650 | 0.971 | 0.001 | 104 (0.16) |
| 4 | 147 | -17129 | 34553 | 35213 | 34746 | 0.970 | 0.500 | 0 (0.00) |

**Table 3.** Model Information for Models with One to Four Classes. *p*LMR = *p*-values for the Lo-Mendell-Rubin likelihood ratio test for K versus K-1 classes. Smallest class frequency = size of the smallest latent class and the relative proportion. N = 658*.*

|  | Unconfident vs. Moderate | Mastery vs. Moderate | Unconfident vs. Mastery |
| --- | --- | --- | --- |
| Intercepts |  |  |  |
| β_0_’s | -1.746* | -0.099 | -1.647* |
| Odds Ratio | 0.174* | 0.906 | 0.193* |
| Native |  |  |  |
| β_1_’s | 0.948* | -0.091 | 1.039* |
| Odds Ratio | 2.581* | 0.913 | 2.826* |
| OGO |  |  |  |
| β_2_’s | 0.306 | 0.284* | 0.021 |
| Odds Ratio | 1.358 | 1.328* | 1.021 |
| ITMA |  |  |  |
| β_3_’s | 0.934* | -0.296* | 1.229* |
| Odds Ratio | 2.545* | 0.744* | 3.418* |
| OGO*ITMA |  |  |  |
| β_4_’s | 0.089 | 0.288* | -0.199 |
| Odds Ratio | 1.093 | 1.334* | 0.820 |

**Table 4.** Predictors of the Latent Class Variable. Odds coefficient and odds ratio of each covariate are reported. The Moderate group was used as the reference for “Unconfident” and “Mastery.” The last column presents information with regard to being in the Unconfident group when the Mastery group was used as the reference group. N = 633. **p* < .01*.*

|  |  | Unconfident | | | | Mastery | |
| --- | --- | --- | --- | --- | --- | --- | --- |
|  | Intercepts | | β’s | Odds Ratio | β’s | | Odds Ratio |
| Interest in STEM Subjects | 3.079* | | -0.872* | - | 0.419* | | - |
| Interest in STEM Activities | 3.225* | | -0.820* | - | 0.415* | | - |
| Retention GPA | 3.173* | | -0.170** | - | 0.289* | | - |
| STEM | 1.016* | | -1.288* | 0.276* | 0.705* | | 2.023* |
| Non-STEM | 0.294 | | 0.375 | 1.455 | 0.304 | | 1.355 |

**Table 5.** Outcomes of the Latent Class Variable. Table reports the unstandardized intercepts, coefficients, and odds ratios by regressing outcomes of interest on the dummy-coded latent class variable. The Moderate group and STEM code = 2 were used as the reference.

**p* < .01 ** *p* = .059

## Figures


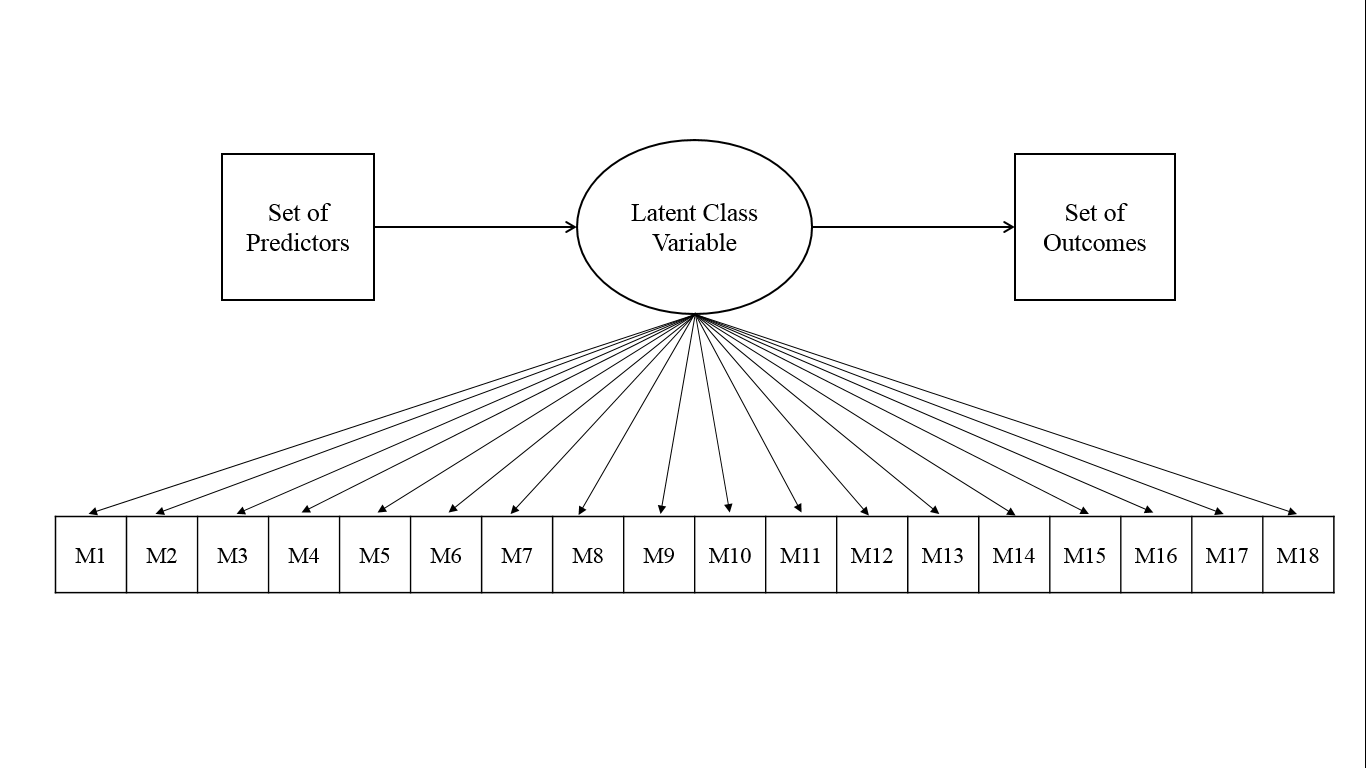


**Figure 1.** Latent profile analysis model with covariates and outcomes.


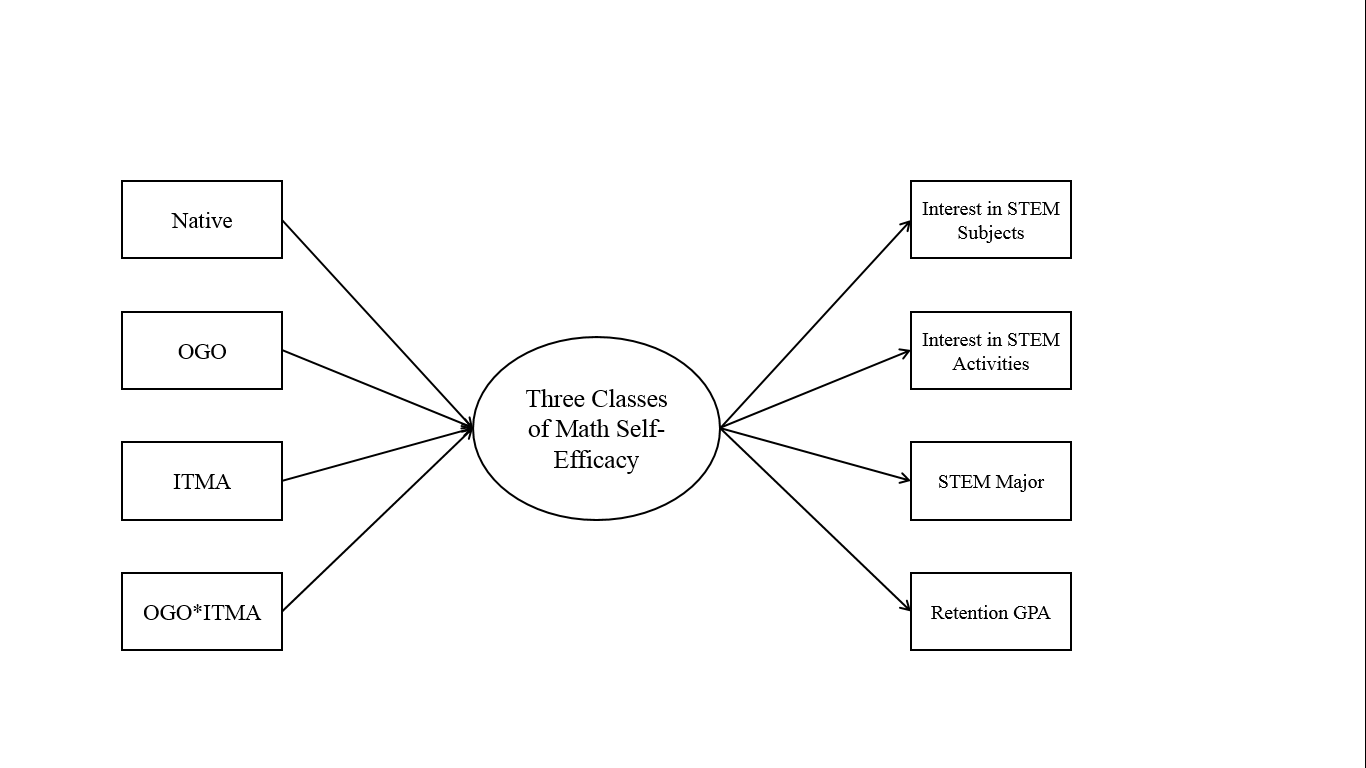


**Figure 2.** Latent profile analysis model that contains the final list of predictors and outcomes.

**Figure 3.** Three-class model estimations for the sources of math self-efficacy measure.

**Figure 4.** The effects of Native, OGO, ITMA, and OGO by ITMA. The Moderate group is used as the reference in this figure. The odds of class membership in the Unconfident group relative to the Mastery group is not presented.

**Figure 5.** The effect of ITMA as a function of OGO. The Moderate group is used as the reference in this figure. The odds of class membership in the Unconfident group relative to the Mastery group is not presented.
